# Dilemmas and choices in healthcare: let’s avoid taking the Ferrari off-road!

**DOI:** 10.1590/S1516-31802009000100002

**Published:** 2009-05-11

**Authors:** Marcos Bosi Ferraz

**Affiliations:** 1 MD. PhD. Professor and Director, Centro Paulista de Economia da Saúde (CPES), Universidade Federal de São Paulo (Unifesp); Director of Medical Economy, Associação Médica Brasileira (AMB); and Director of Institutional Relations, Grupo Fleury, São Paulo, Brazil. E-mail: marcos.ferraz@fleury.com.br

First of all, I would like to thank Professor Álvaro Atallah for bringing this issue for discussion. High-quality debate is a very powerful strategy for stimulating our colleagues to think about key healthcare topics and for sensitizing healthcare professionals and even the lay public regarding the importance of recognizing the challenges and dilemmas we face when making healthcare decisions.

I invite all readers of this journal to critically appraise the concepts and the way of thinking about healthcare that are presented in the recently published book “Dilemas e Escolhas do Sistema de Saúde”.^1^

There is a possible misunderstanding about the importance and feasibility of implementing health technology assessments in Brazil that I would like to better clarify. Health technology assessments have been conducted for decades in several regions of the world, including Brazil. What changed recently (maybe 20 to 30 years ago, to be more specific) is the recognition that healthcare decisions are becoming more and more complex but, on the other hand, they are becoming more structured and based on methods that are fairly universally accepted or recognized. With the ever increasing investments in scientific development, discoveries in the field of biological sciences, the globalization process (thanks to information and communication technologies) and increasing education levels among the public (fortunately), the options now available for improving the health of the community are huge.

Unfortunately, the speed and amount of economic growth and development among nations worldwide have not uniformly followed this trend. As a result, choices have to be made, because the opportunities to do more good than harm are greater than the ability of nations to satisfy the perceived healthcare needs of their populations. In this sense, tough decisions have to be made everyday everywhere: in other words, decisions that may profoundly affect the lives of individual citizens are made everyday. I would generally say that decisions involving “YES” are not hard to make, but the ones that involve saying “NO” to some interventions, strategies or healthcare programs are really the most difficult ones, especially when they are recognized to add quality of life or quantity of life to individuals or communities. Issues regarding the efficiency of healthcare systems and their equity cannot be dissociated. In a country like ours, it is very easy to have decisions (lots of yeses) that it is unfeasible to implement, just because the healthcare system is still unprepared for them, As an illustration, the system lacks infrastructure and qualified human resources, or fails to identify and recognize specific interests that are more valued in the decision process than are the interests of society, the community or citizens themselves. Moreover, such decisions may not be available to everyone who needs them.

Health technology assessments and decisions to incorporate new technologies into the system (or restrict the use of old and already available technologies) do not solely involve exercising evidence-based medicine or healthcare. They also involve firstly the definition, recognition and ranking of healthcare priorities and, additionally, the appraisal of healthcare organizations (especially the infrastructure available) and the ethical, economic and social issues. Even considering evidence-based medicine or healthcare, it is important to mention that the appraisal should value community effectiveness, which takes into account issues such as the access of individuals (patients) to the healthcare system, the ability to diagnose correctly the target disease (diagnostic and screening accuracy), the effectiveness of interventions and the compliance of providers and patients (or individuals). I am confident that, even in developed nations, there is still not enough good quality data regarding the community effectiveness of most interventions.

Furthermore, it is crucial to emphasize that healthcare systems are very complex systems, and that health technology assessment is a new field or discipline within biological sciences that is also tremendously complex and still under development. As mentioned earlier, evidence-based medicine or evidence-based healthcare is just part of it (although a very important part). It is important also to recognize that biological science is characterized by transitory truths, and that new knowledge usually modifies some temporary truths.

Ideally, decisions are (or should) usually be based on evidence (the knowledge accepted to be the best estimate of the truth) and the expression of society’s preferences and values. Sometimes, the knowledge (evidence) is available and there is no question about its validity and applicability within a defined environment. However, society’s preferences and values do not follow the same logic. One very simple example is the issue of smoking in our society: there is plenty of evidence that it does more harm than good but, in spite of that, societal preferences and values and the willingness to respect personal decisions overrule the most logical and sensible decision. As I mention in my book,^1^ and especially true regarding this example, “individual decisions affect the community and community decisions affect and restrict individual decisions”. The process of making choices is, therefore, much more complex than it appears. Health technology assessment is a live example of the complexities of the healthcare system. I would like also to quote a famous phrase by an American journalist, H. L. Menken, that clearly expresses the challenge we face in healthcare when making decisions: “To every complex question there is a simple answer...and it is wrong”. Let’s avoid simple and quick answers involving our healthcare system!

To conclude, and also to stimulate readers to think about the challenges we face every day in healthcare, I would generally say that nowadays, we sometimes appear to have the truth in our hands (taking the nature of the biological sciences into consideration), but we forget to consider the limitations that exist regarding making healthcare readily available, considering the perceived needs of our citizens and populations, as well as the infrastructure available to enable these strategies, interventions and policies to achieve the desired goals. By this, I mean that healthcare decisions should be made very responsibly and should recognize the economic constraints we face, the healthcare system we have and the preferences and values of our society. Efficiency and equity should be always considered in any healthcare decision. Such decisions obviously should consider all the methodological rigor implied or exercised in evidence-based medicine, but they should go far beyond it. To make this point, I customarily say to my colleagues: “sometimes the challenge is not to show that a Ferrari is an excellent car and does more good than harm (lots of evidence available) for most people (even taking into account and respecting the possible distinct preferences and values); the challenge is to avoid taking the Ferrari off-road!”.
